# Defective *Slc7a7* transport reduces erythropoietin compromising erythropoiesis

**DOI:** 10.1186/s10020-025-01100-0

**Published:** 2025-01-29

**Authors:** Judith Giroud-Gerbetant, Fernando Sotillo, Gonzalo Hernández, Irene Ruano, David Sebastián, Joana Fort, Mayka Sánchez, Günter Weiss, Neus Prats, Antonio Zorzano, Manuel Palacín, Susanna Bodoy

**Affiliations:** 1 Institute for Research in Biomedicine (IRB Barcelona), The Barcelona Institute of Science and Technology (BIST), Barcelona, Spain; 2 Centro de Investigación Biomédica en Red Enfermedades Raras (CIBERER), Instituto de Salud Carlos III, Madrid, U-731 Spain; 3 Department of Basic Sciences, Iron Metabolism: Regulation and Diseases Group. Faculty of Medicine and Health Sciences, Universitat Internacional de Catalunya (UIC), Sant Cugat, Spain; 4 Department of Biochemistry and Physiology, School of Pharmacy and Food Sciences, Universitat de Barcelona, Barcelona, Spain; 5 Department of Internal Medicine II (Infectious Diseases, Immunology, Rheumatology and Pneumology), Medical University of Innsbruck, Innsbruck, Austria; 6 Departament de Bioquímica i Biomedicina Molecular, Universitat de Barcelona (UB), Barcelona, Spain; 7 Centro de Investigación Biomédica en Red Diabetes y Enfermedades Metabólicas (CIBERDEM), Madrid, Spain; 8 Biosciences Department, Faculty of Sciences, Technology and Engineering, Universitat de Vic – Universitat Central de Catalunya (UVic-UCC), Vic, Spain; 9 Institute of Biomedicine of the University of Barcelona (IBUB), Barcelona, Spain

**Keywords:** Rare disease, Amino acids, Erythropoiesis, Kidney disease

## Abstract

**Background:**

Lysinuric protein intolerance is a rare autosomal disorder caused by mutations in the *Slc7a7* gene that lead to impaired transport of neutral and basic amino acids. The gold standard treatment for lysinuric protein intolerance involves a low-protein diet and citrulline supplementation. While this approach partially improves cationic amino acid plasma levels and alleviates some symptoms, long-term treatment is suggested to be detrimental and may lead to life-threatening complications characterized by a wide range of hematological and immunological abnormalities. The specific cause of these hematopoietic defects—whether intrinsic to hematopoietic cells or driven by external factors—remains unclear. Given the limitations of current citrulline-based treatments and the unknown role of SLC7A7 in red blood cell production, there is an urgent need to investigate the pathways affected by SLC7A7 deficiency.

**Methods:**

We employed total inducible and cell type-specific *Slc7a7* knockout mouse models to determine whether the hematological abnormalities observed in LPI are due to the loss of *Slc7a7* function in hematopoietic cells. We analyzed erythropoiesis in these mice and performed bone marrow transplantation experiments to assess the role of *Slc7a7* in erythroblasts and myeloid cells. The statistical significance of differences between groups was evaluated via standard statistical tests, including Student’s *t* test and ANOVA.

**Results:**

Whole-body *Slc7a7* knockout mice presented impaired erythropoiesis. However, this defect was not replicated in mice with *Slc7a7* deficiency restricted to erythroblasts or myeloid cells, suggesting that the observed hematopoietic abnormalities are not due to intrinsic *Slc7a7* loss in these cell types. Additionally, bone marrow transplants from control mice did not rescue the hematopoietic defects in *Slc7a7*-deficient mice, nor did the transplantation of *Slc7a7*-deficient cells induce defects in control recipients. Further investigation indicated that defective erythropoiesis is linked to impaired erythropoietin production in the kidney and subsequent iron overload.

**Conclusions:**

The hematopoietic defects in the Lysinuric protein intolerance mouse model are not caused by intrinsic *Slc7a7* loss in hematopoietic cells but rather by impaired erythropoietin production in the kidney. This finding opens potential avenues for therapeutic strategies targeting erythropoietin production to address hematological abnormalities in humans with lysinuric protein intolerance.

**Supplementary Information:**

The online version contains supplementary material available at 10.1186/s10020-025-01100-0.

## Introduction

Lysinuric protein intolerance (LPI; MIM 222700) is a rare autosomal recessive disorder that was first discovered in Finland, with clusters of cases identified worldwide since the 1980s (Simell 2001). LPI is caused by mutations in the *Slc7a7* gene (Torrents et al. 1999; Borsani et al. 1999) located on chromosome 14q11.2, which encodes y^+^LAT1. Together with the heavy subunit 4F2hc (also named CD98h or SLC3A2), y^+^LAT1 forms a heteromeric amino acid transporter essential for the transport of cationic amino acids (CAAs), such as arginine, lysine, and ornithine (Torrents et al. 1998). This impaired transport leads to a wide array of metabolic and immunological alterations, including hyperexcretion of the CAA, disruption of the urea cycle, and chronic anemia (Alqarajeh et al. 2020), with some cases progressing to chronic kidney disease (Ogier de Baulny et al. 2012; Tanner et al. 2007; IJzermans et al. 2022; Estève et al. 2017). Despite its diverse clinical manifestations, no targeted cure for LPI exists, largely owing to its complex and variable disease progression (Ogier de Baulny et al. 2012).

The current standard treatment for patients with LPI involves a low-protein diet combined with citrulline supplementation, which partially restores urea cycle function by bypassing defective amino acid transport. Citrulline is converted to arginine, helping reduce hyperammonaemia; however, studies have indicated that, in some cases, high doses of citrulline may paradoxically worsen disease progression by promoting excess nitric oxide production, potentially leading to side effects such as vasodilation and blood pressure instability (Contreras et al. 2021; Noguchi and Takahashi 2019). These limitations highlight the need for new therapeutic approaches that more effectively manage the metabolic and hematological complications of LPI.

The y^+^LAT1/4F2hc transporter exchanges CAA for neutral amino acids in the presence of sodium. The heterodimer is located in the basolateral membrane of kidney and intestinal epithelial cells, as well as in certain nonpolarized cells, such as macrophages, monocytes, and erythrocytes (Torrents et al. 1998; Fotiadis et al. 2013). Different lines of evidence underscore the pivotal role of *Slc7a7* in the regulation of hematopoietic processes, as LPI patients often present with chronic microcytic normochromic anemia, which suggests disruptions in iron metabolism and impaired hemoglobin synthesis (Contreras et al. 2021). Furthermore, these patients often experience recurrent respiratory infections, a predisposition to autoimmune diseases, and, in severe cases, life-threatening varicella infections, all of which are suggestive of compromised immune function (Contreras et al. 2021; Lukkarinen et al. 1999, 1998; Zhang and Cao 2017; Parto et al. 1994). In this context, SLC7A7 has also been reported to play a significant role in cationic amino acid transport in alveolar macrophages (Rotoli et al. 2007), potentially contributing to surfactant recycling (Barilli et al. 2012). However, its effects on hematopoietic cell differentiation and function remain underexplored.

In this study, we used *Slc7a7* knockout mice, a model that closely mimics LPI disease (Bodoy and Sotillo 2019), to investigate the impact of SLC7A7 on hematopoietic lineages. Our analysis revealed that *Slc7a7* knockout mice exhibit significant defects in blood cell parameters, including markedly reduced mean corpuscular volume and a trend toward diminished mean corpuscular hemoglobin and erythrocyte hemoglobin content, despite normal red blood cell counts. Interestingly, these defects were not observed when *Slc7a7* was specifically deleted in erythroblasts or myeloid cells, suggesting that hematological abnormalities are not intrinsic effects of SLC7A7 deficiency in these cells. Instead, our findings suggest that kidney damage resulting from SLC7A7 dysfunction decreases erythropoietin production, leading to impaired erythropoiesis and iron accumulation. These insights shed new light on the mechanisms underlying hematological abnormalities in LPI and suggest that administering exogenous erythropoietin could be a promising therapeutic strategy to alleviate these complications.

## Materials and methods

### Animals

All animal work was conducted following established guidelines. The project (DARP No. 9177) was favorably assessed by the Institutional Animal Care and Use Committee of the Parc Científic de Barcelona (IACUC-PCB), and the IACUC considered that the project complied with standard ethical regulations and met the requirements of current applicable legislation (RD 53/2013 Council Directive; 2010/63/UE; Order 214/1997/GC). Further details are specified in the *Supplementary methods*.

## Methods details

### Flow cytometry and cell sorting

For the analysis of splenocytes and bone marrow (BM) cells, the spleens were crushed, and the BM was extracted by flushing the bones. Both tissues were then filtered through a 40-μm cell strainer to obtain a single-cell suspension. Splenocytes and bone marrow cells were incubated with Fc-blocking (anti-mouse CD16/32; Thermo Fisher) for 15 min on ice.

For flow cytometry, splenic cells were stained with APC-conjugated CD44 and FITC-conjugated Ter-119 antibodies from BioLegend for 30 min on ice. Flow cytometry analysis was performed using an Aurora flow cytometer from Cytek Biosciences.

For FACS analysis of BM cells, samples were stained with APC-conjugated CD44, FITC-conjugated Ter-119, and PE-conjugated CD71 from BD Pharmingen for 30 min on ice. For *Slc7a7* expression in alveolar macrophages, red pulp macrophages, bone marrow macrophages, splenocytes and single-cell suspensions from BM were stained with PE-conjugated F4/80 and APCCy7-conjugated Cd11b. Cell sorting to achieve a purity greater than 90% was conducted via a FACS Fusion II instrument from BD Biosciences. The analysis excluded doublets, and where possible, dead cells were identified and excluded via DAPI staining.

Red blood cells (RBCs) were stained separately with PE-conjugated CD47, and approximately 1 × 10^6^ erythrocytes were incubated with anti-CD47-PE for 30 min on ice. To assess apoptosis in erythrocytes, 1 × 10^6^ RBCs were resuspended in 1X Annexin V binding buffer (Invitrogen). Each sample was then stained with 5 µL of Annexin V and incubated for 15 min at room temperature. Following incubation, the RBCs were centrifuged, the supernatant was discarded, and the cells were resuspended in FACS buffer containing propidium iodide for subsequent analysis.

Data from both flow cytometry and cell sorting were analyzed via FlowJoTM software.

### Bone marrow-derived macrophages

BM cells from 12-week-old mice were flushed from the femurs and tibiae. The cell suspension was lysed for 5 min in erythrolysis buffer (R&D Systems) at 4 °C, washed, resuspended, and cultured for 7 days in DMEM supplemented with 10% heat-inactivated fetal bovine serum, 50 U/mL penicillin, 50 μg/mL streptomycin and 30% L-cell-conditioned medium. Six days after seeding, the cells were harvested and reseeded with the previously mentioned conditioned medium for 24 h.

### Respirometry studies

Respirometry studies were performed on freshly isolated mitochondrial extracts from the kidney cortex via high-resolution respirometry (Oroboros Oxygraph-2 k, Oroboros Instruments) as previously described (Sebastián et al. 2012).

### Tissue iron content

The nonheme iron content in liver and spleen tissues was quantified via a bathophenanthroline colorimetric assay. The tissue samples were first dried at 45 °C for 72 h and then accurately weighed. For iron extraction, the dried tissues were digested in a 10% trichloroacetic acid/10% hydrochloric acid solution at 65 °C for 48 h to ensure complete deproteinization. After dilution, the extracts were treated with 0.01% bathophenanthroline disulfonic acid, 0.1% thioglycolic acid, and a 7 M sodium acetate solution to develop the colorimetric reaction. The absorbance was measured at 535 nm via an Ultrospec 3100pro spectrophotometer (Amersham Biosciences). Iron concentrations in the samples were calculated by interpolating values from a standard curve and normalizing them to the weight of the dried tissue.

### Mouse whole blood count and serum analysis

Blood was collected via cardiac puncture directly into EDTA-coated microtubes to prevent coagulation. To analyze the erythrocyte area, fresh samples of EDTA-anticoagulated blood were used to prepare blood smears. The smears were then stained via the Diff‒Quik staining method to increase the visibility of the erythrocytes. For each mouse, 10 images were acquired at 40 × magnification to ensure a representative analysis. The stained images were processed via ImageJ software to assess the erythrocyte area, allowing for the quantitative measurement of cell size across samples.

Serum albumin, total protein, blood urea nitrogen, lactate dehydrogenase and total bilirubin were analyzed via a veterinary clinical chemistry analyzer (Pointcare cV3 (RAL)) via a general panel (MN00001).

### Red blood cell hemolysis

For analysis of hemolysis under osmotic stress, freshly isolated RBCs from the control and experimental groups were subjected to increasing concentrations of sodium chloride (NaCl). Blood samples were collected in EDTA tubes and centrifuged at 1500 × g for 10 min to separate the plasma and buffy coat. The packed RBCs were washed three times in PBS and then resuspended in isotonic PBS to achieve a 5% hematocrit. This RBC suspension was then incubated with NaCl solutions prepared at concentrations ranging from 0.1% to 0.9%. Each concentration was tested in triplicate. After a 30-min incubation at room temperature, the samples were centrifuged at 2000 × g for 10 min, and the absorbance of the supernatant was measured at 540 nm via a spectrophotometer to determine the degree of hemolysis. The percentage of hemolysis was calculated relative to a positive control (water), which was assumed to result in complete lysis of erythrocytes. The data were then used to plot a hemolysis curve, illustrating the osmotic fragility of the RBCs under different salt concentrations.

### Analysis of reactive oxygen species production in erythrocytes using CM-H2DCFDA

To measure the intracellular reactive oxygen species (ROS) levels in erythrocytes, the fluorescent probe CM-H2DCFDA (5-(and-6)-chloromethyl-2′,7′-dichlorodihydrofluorescein diacetate, acetyl ester) was utilized. Freshly isolated erythrocytes were washed three times in cold PBS to remove the plasma and buffy coat. The washed erythrocytes were then resuspended in PBS to a final concentration of 1 × 10^6^ cells/mL. CM-H2DCFDA was added to the erythrocyte suspension at a final concentration of 10 µM, and the mixture was incubated at 37 °C for 30 min in the dark to allow complete loading of the probe. After incubation, the cells were washed twice with cold PBS to remove any unbound probe and resuspended in PBS for immediate analysis.

Flow cytometric analysis was performed via a flow cytometer (FACS Fusion II) to detect the fluorescence emission at 530 nm, which is indicative of ROS production within the cells. The oxidation of CM-H2DCFDA to its fluorescent form reflects the level of cellular ROS. The mean fluorescence intensity of the oxidized probe in the erythrocytes was quantified via FlowJo software, which provides a measure of the oxidative stress within the samples.

### Reticulocyte counts

Fresh EDTA-anticoagulated blood samples were stained with new methylene blue dye (Sigma, 556,416) following the manufacturer’s instructions. Films on glass slides were then performed for each sample. New methylene blue was used to evaluate aggregates of ribosomes and mitochondria in reticulocytes. The percentage of reticulocytes was calculated as follows:

$$\% {\text{Reticulocytes}} = \frac{{\text{Nb of reticulocytes}}}{{1000 {\text{RBCs}}}} x 100$$

### Total kidney erythropoietin measurement

Kidneys were perfused with Hank’s balanced salt solution, and total kidney extract was obtained by mechanical digestion with PBS supplemented with a protein inhibitor (Protease Inhibitor Cocktail Set III, Merck). Erythropoietin levels were subsequently determined via commercial enzyme-linked immunosorbent assay kits (R&D Systems, MEP00B).

### Statistical analysis

The data were analyzed via GraphPad Prism Version 8 software. Statistical analysis was performed via Student’s *t* test and ANOVA, as specified in each figure legend.

## Results

### Slc7a7 knockout mice display defective erythropoiesis

**Fig. 1:**
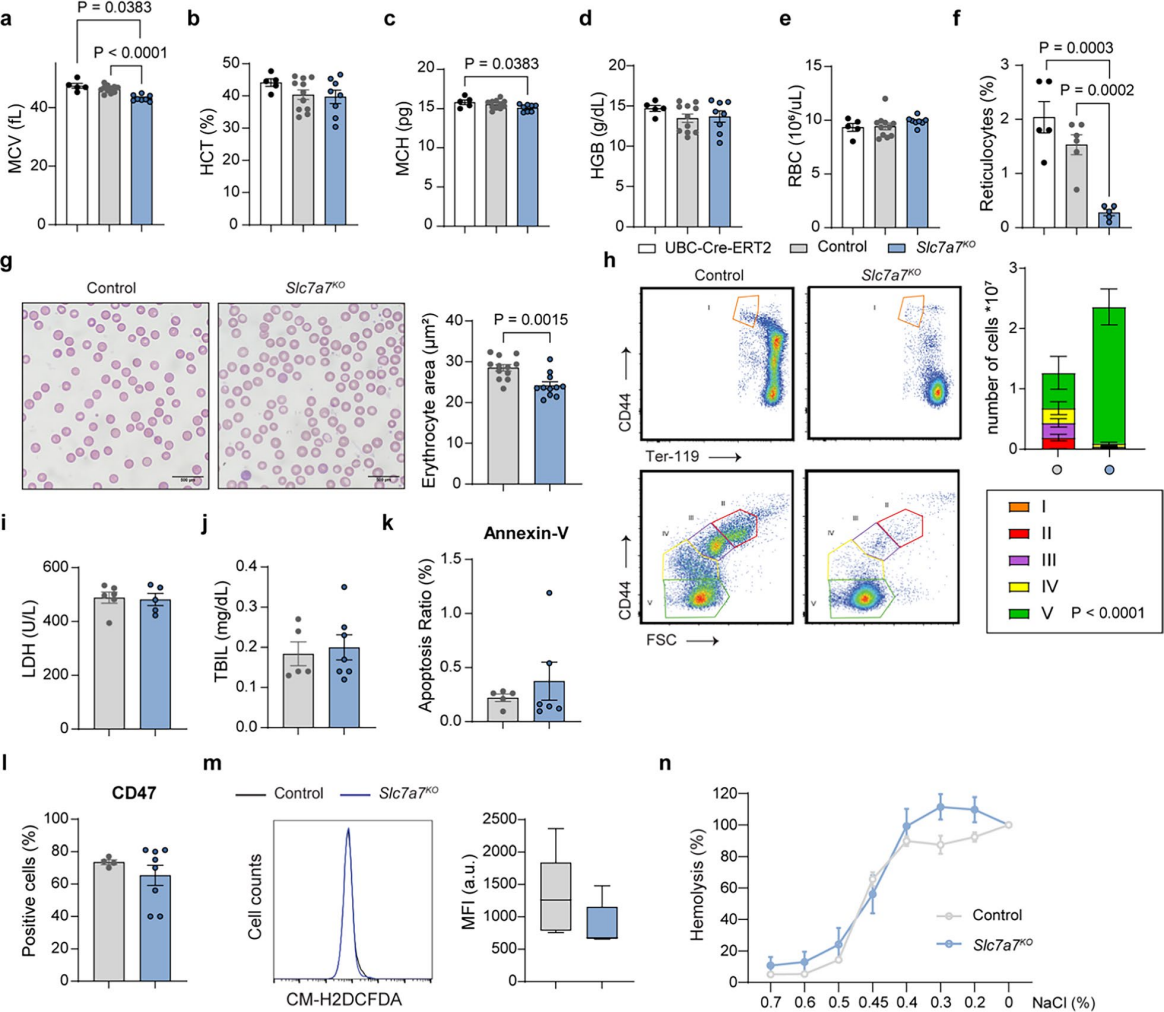
Impaired erythropoiesis and red blood cell fitness in *Slc7a7* knockout mice. **a**–**f** Complete blood count analysis of only UBC-Cre-ERT2, control and *Slc7a7*^*KO*^ mice after 10 days on a low-protein diet. Hemoglobin (Hb), hematocrit (Hct), mean corpuscular hemoglobin (MCH), mean corpuscular volume (MCV), red blood cell count (RBC) and reticulocyte count are shown. **g** Representative brightfield images from three independent experiments of blood smears from control and *Slc7a7*^*KO*^ mice. The quantification of the erythrocyte area is presented in the adjacent bar graph. **h** Flow cytometry analysis of bone marrow erythropoiesis. Bone marrow cells were labeled with antibodies against CD44 and Ter-119 to identify erythroid progenitors across five maturation stages (I–V). *Slc7a7*^*KO*^ mice presented a significant increase in Stage V precursors (*P* < 0.0001), whereas Stages I–IV were not significantly different, although there is a noticeable trend towards reduction. The data are presented as the means ± SEMs and were analyzed via two-way ANOVA. Representative results from four independent experiments are shown. **i** Lactate dehydrogenase and **j** total bilirubin levels in the serum of control and *Slc7a7*^*KO*^ mice. **k** Annexin-V and **l** CD47 levels in RBCs from control and *Slc7a7*^*KO*^ mice. **m** Mean fluorescence intensity of RBCs from control and *Slc7a7*^*KO*^ mice in response to CM-H2DCFDA. **n** Percentages of hemolysis in control and *Slc7a7*^*KO*^ mice in response to increasing concentrations of NaCl (0–0.7%). Unless specified, all the data are presented as the means ± SEMs. Statistical analysis was performed via a two-tailed unpaired Student’s *t* test. Each data point represents an individual animal

Lysinuric protein intolerance (LPI) in humans has been associated with microcytic and normochromic anemia (Estève et al. 2017; Contreras et al. 2021; Mauhin et al. 2017). To investigate the underlying mechanisms, we examined a mouse model with ubiquitous *Slc7a7* ablation (*Slc7a7*^fl/fl^ and UBC-Cre-ERT2 mice, herein referred to as *Slc7a7* knockout mice) (Bodoy and Sotillo 2019) and compared them with control mice (*Slc7a7* LoxP/LoxP without the UBC-Cre-ERT2 protein). At 12 weeks of age, the mice were fed a tamoxifen diet for 7 days. The mice were then maintained on a low-protein diet for 20 days, resulting in a 50% survival rate in those lacking *Slc7a7* (Bodoy and Sotillo 2019). Blood analysis revealed significantly lower mean corpuscular volume, reduced hematocrit, and a trend toward lower mean corpuscular hemoglobin and erythrocyte hemoglobin content, although red blood cell (RBC) counts remained unchanged (Supplementary Table 1). Owing to high mortality after 20 days, we shortened the low-protein diet duration to 10 days, which resulted in a 100% survival rate. All subsequent experiments were conducted using this adjusted timeframe. Under this adjusted protocol, the mean corpuscular volume reduction was the most prominent change, likely reflecting an early effect of *Slc7a7* ablation (Fig. 1a–e). To rule out a possible confounding effect of the tamoxifen diet on the expression of the Cre protein, we also used UBC-Cre-ERT2 mice. Control studies confirmed that these effects were not due to tamoxifen-induced Cre expression (Fig. 1a–f).

Next, we evaluated erythrocyte size through histological analysis of peripheral blood, which revealed a reduced erythrocyte area in *Slc7a7* knockout mice (Fig. 1g), along with fewer circulating reticulocytes (Fig. 1f), indicating possible impaired erythropoiesis (Klei et al. 2017). When erythrocyte maturation markers were examined, we observed that *Slc7a7* knockout mice presented a tendency to diminished absolute numbers of erythroblast precursors at stages II-IV (per femur and tibiae). In contrast, mature erythroid precursors (stage V) were significantly enriched (Fig. 1h). This striking defect in early-stage precursors was confirmed via the use of two independent markers, CD44 (Fig. 1h) and Cd71 (Chen et al. 2009), both of which have similar outcomes (Supplementary Fig. 1a). Reduced RBC production is often accompanied by iron accumulation, as erythropoiesis is one of the most iron-demanding processes in organisms (Muckenthaler et al. 2017). Similarly, *Slc7a7* knockout mice presented significantly increased iron deposits in the liver and spleen, as observed through Perls Prussian blue staining and direct spectrophotometric analysis of tissue biopsies (Supplementary Fig. 1b-d).

To investigate whether reduced erythropoiesis stemmed from increased RBC destruction, we measured the serum lactate dehydrogenase and total bilirubin levels, both of which were comparable between control and *Slc7a7* knockout mice (Fig. 1i-j), ruling out hemolytic anemia. Additionally, Annexin-V and Cd47 analyses of markers associated with RBC clearance (Boas et al. 1998; Burger et al. 2012), representing “eat-me” and “don’t-eat-me” signals, respectively, revealed no significant differences in RBC fitness between genotypes (Fig. 1k-l). Furthermore, reactive oxygen species production was similar across genotypes in freshly isolated RBCs, and no differences were observed in RBC osmotic fragility upon exposure to increasing NaCl concentrations, indicating comparable membrane stability (Fig. 1m-n). Collectively, these findings suggest that the reduced number of RBC precursors in *Slc7a7* knockout mice is not attributable to decreased RBC survival or fitness.

In adult mice, erythropoiesis occurs predominantly in the bone marrow. However, in the event of defective erythropoiesis, the spleen can take over erythroid production (Cenariu et al. 2021). Histological analyses indicated that control and *Slc7a7* knockout mice presented minimal levels of extramedullary erythropoiesis in the red pulp of the spleen. Even if a decrease in the size and cellularity of the red pulp was detected (Supplementary Fig. 1e), flow cytometry analysis indicated that *Slc7a7* knockout mice did not show signs of compensatory erythropoiesis under basal conditions or after acute hemolytic anemia caused by phenylhydrazine (Supplementary Fig. 1f–g).

### Loss of Slc7a7 in myeloid and erythroblast cell lineages has a minimal effect on bone marrow erythropoiesis

**Fig. 2:**
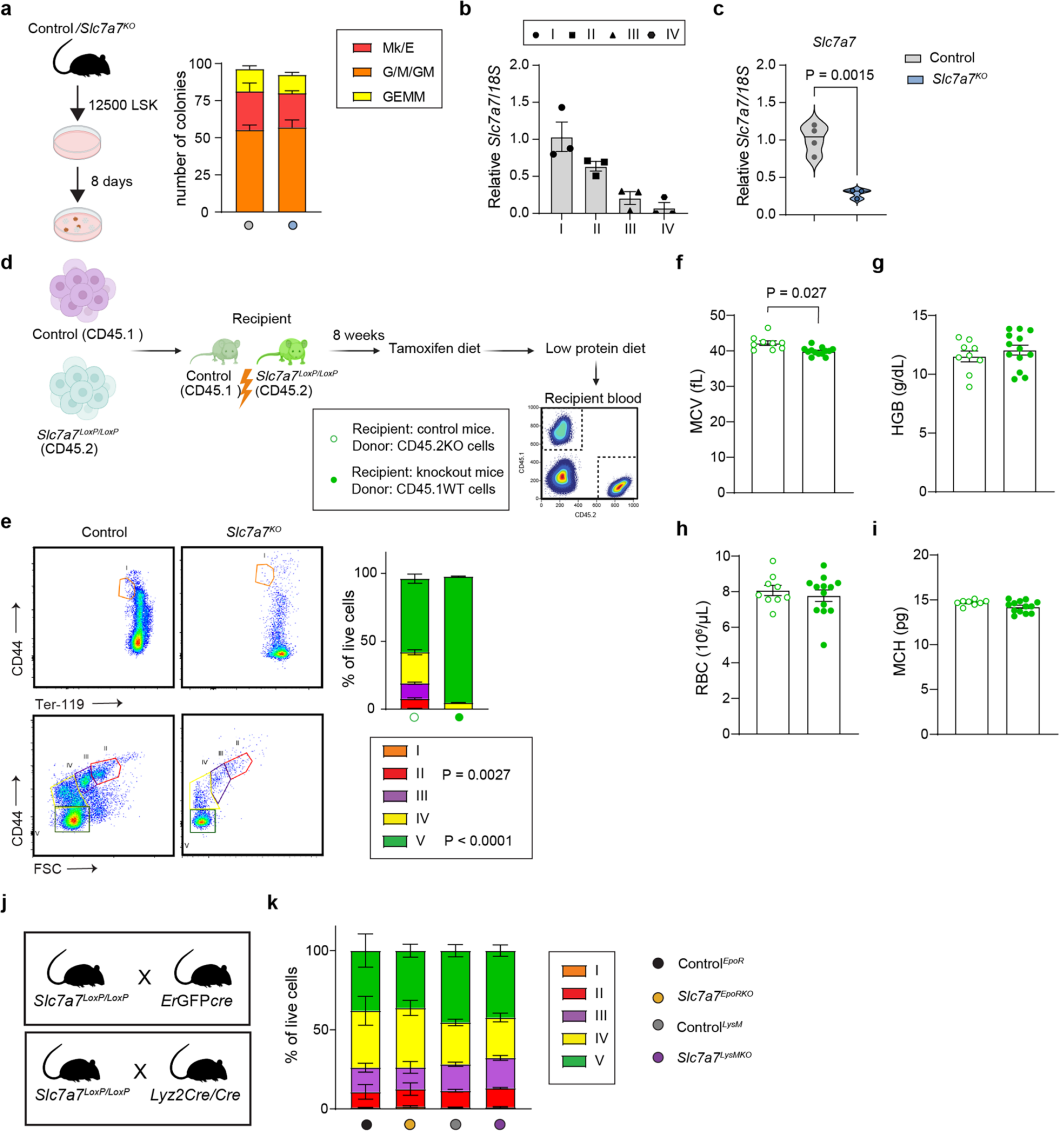
*Slc7a7* intrinsic expression does not drive deficient erythropoiesis in *Slc7a7* knockout mice. **a** Colony-forming unit assays of 12,500 Lin^−^Sca-1^+^c-Kit^+^ cells isolated from control and *Slc7a7*^*KO*^ mice. The colony types included granulocyte/monocyte (G/M/GM), granulocyte (G), monocyte (M), megakaryocyte/erythroid (Mk/E), and mixed granulocyte-erythroid-monocyte-megakaryocyte (GEMM) CFUs (n = 4). **b** Real-time PCR analysis of *Slc7a7* expression in sorted erythroid progenitors (highlighting stage-specific expression I–IV) from control mice. **c** Real-time PCR analysis of *Slc7a7* expression across erythroid progenitor stages (IV, III, II, I) in control and *Slc7a7*^*KO*^ mice. **d** Schematic representation of bone marrow transplantation in control (CD45.1) and *Slc7a7*^*KO*^ (CD45.2) mice. The experimental setup involves transplantation of wild-type cells into *Slc7a7*^*KO*^ recipients and vice versa, followed by tamoxifen and a low-protein diet. **e** Left: Representative dot plots showing the gating strategy for erythroid progenitors in bone marrow, identifying five clusters (I–V). Right: Quantification of erythroid progenitor populations. Clusters I–III showed no significant differences, whereas reductions were observed in Cluster II (*P* = 0.0027) and Cluster V (*P* < 0.0001) in *Slc7a7*^*KO*^ mice. Representative data from three independent experiments are shown. Statistical analysis was performed via two-way ANOVA. **f** Complete blood count analysis of the transplanted mice. *Slc7a7*^*KO*^ recipients transplanted with wild-type cells (filled circles) and wild-type recipients transplanted with *Slc7a7*^*KO*^ cells (empty circles) presented significant differences in the mean corpuscular volume (MCV, *P* = 0.027) but no significant changes in HGB, RBC, or MCH. **j** Schematic representation of *Slc7a7*-specific ablation in myeloid cells via Lyz2Cre (*Slc7a7*^*LysM*^) and erythroid cells via ErGFPCre (*Slc7a7*^*EpoR*^). **k** Quantification of erythroid precursor populations (Stages I–V) in *Slc7a7*^*LysMKO*^, *Slc7a7*^*EpoRKO*^, and their respective control mice. The data are from n = 7 (Lyz2Cre) and n = 3 (EpoR) mice. Statistical analysis was performed via two-way ANOVA. Unless specified, all the data are presented as the means ± SEMs. Statistical analysis was performed via a two-tailed unpaired Student’s *t* test. Each data point represents a single animal

Given the pronounced erythroid maturation arrest in *Slc7a7* knockout mice, we sought to understand the role of *Slc7a7* in erythrocyte differentiation and function. We first evaluated the viability and differentiation potential of hematopoietic stem cells from both control and knockout mice via the use of sorted Lin^−^Sca1^+^cKit^+^ cells in colony-forming unit assays. The results revealed no significant differences between genotypes in their capacity to generate erythrocytes or other hematopoietic lineage cells, such as granulocytes or monocytes (Fig. 2a). Next, we determined that in control mice, *Slc7a7* is expressed in erythroid precursors and its expression decreases as maturation progresses (Fig. 2b). We then investigated whether *Slc7a7* expression was significantly diminished in *Slc7a7* knockout mice. Gene expression analysis revealed a dramatic reduction of approximately 90% in *Slc7a7* levels in erythrocyte precursors (Ter119^+^ sorted population) from *Slc7a7* knockout mice (Fig. 2c).

The preservation of cell viability and differentiation ex vivo led us to investigate whether the defects observed in the whole-body knockout models stemmed from intrinsic or extrinsic factors. To address this, we employed a bone marrow transplant strategy in which bone marrow cells from wild-type (CD45.1^+^) mice were transplanted into irradiated *Slc7a7* knockout (CD45.2^+^) mice, while *Slc7a7* knockout bone marrow cells (CD45.2^+^) were transplanted into irradiated wild-type mice (CD45.1^+^). At 8 weeks posttransplantation, a tamoxifen diet was administered for 1 week, after which the mice were maintained on a low-protein diet for 10 days before blood parameters were examined (Fig. 2d, Supplementary Fig. 2a-b). *Slc7a7*-deficient hematopoietic cells presented normal erythopoietic differentiation profiles and mean corpuscular volume when they were transplanted into wild-type mice (Fig. 2e–i). These results suggest that deficiency in erythroid precursors is driven by extrinsic factors. In support of this hypothesis, wild-type cells transplanted into the *Slc7a7* knockout mice did not improve the erythropoiesis defects.

To further confirm that the erythropoiesis defect arises from an extrinsic factor, we generated two independent knockout mouse models that specifically target *Slc7a7* expression in the erythroid and myeloid lineages. The first relies (*Slc7a7*^*EpoRKO*^) on the use of the erythropoietin receptor promoter to drive Cre recombinase expression specifically in erythroid progenitors (ErGFPCre) (Heinrich et al. 2004), whereas the second (*Slc7a7*^*LysMKO*^) uses the lysozyme 2 promoter (Lyz2Cre) (Clausen et al. 1999) and targets the whole myeloid cell lineage, including erythrocytes (Fig. 2j). Analysis of these models revealed a 95% reduction in *Slc7a7* transcript levels in the erythroid progenitors of *Slc7a7*^*EpoRKO*^ mice (Supplementary Fig. 2c). Furthermore, both *Slc7a7* transcript and protein expression in the bone marrow-derived macrophages of *Slc7a7*^*LysMKO*^ mice were effectively ablated, confirming the successful knockout of *Slc7a7* in myeloid cells (Supplementary 2d and 2f). In line with the results of the bone marrow transplant experiments, *Slc7a7*^*EpoRKO*^ and *Slc7a7*^*LysMKO*^ mice presented erythroid maturation processes similar to those of control mice (Fig. 2k, Supplementary Fig. 2e). These results collectively suggest that *Slc7a7* plays a minimal intrinsic role in red RBC maturation, reinforcing the hypothesis that erythropoiesis defects in *Slc7a7* knockout models are driven primarily by extrinsic factors.

### Reduced circulating erythropoietin levels are associated with impaired erythropoiesis and diminished kidney function

**Fig. 3:**
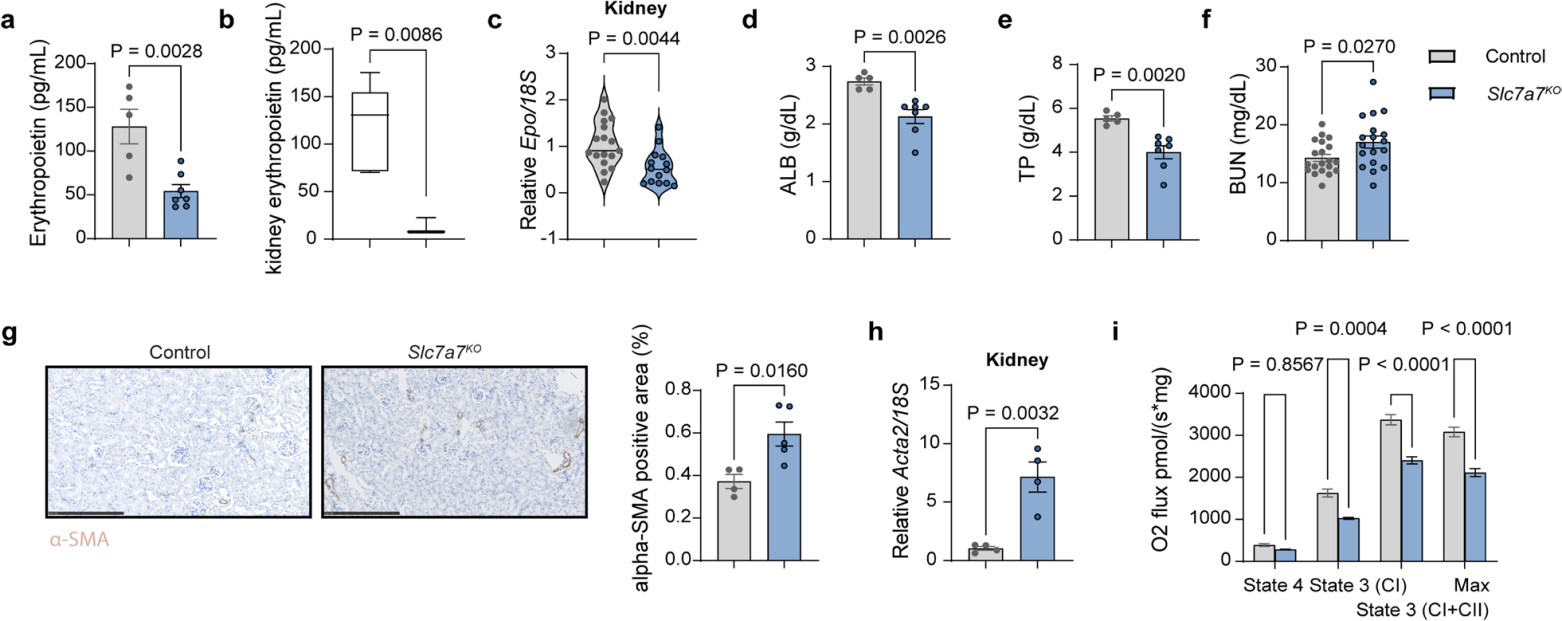
*Slc7a7* knockout mice exhibit impaired kidney function associated with reduced erythropoietin levels and metabolic dysfunction. **a** Circulation of erythropoietin serum levels and **b** kidney erythropoietin content in control and *Slc7a7*^*KO*^ mice (blue). **c** Real-time PCR analysis of *Epo* transcript levels in total kidneys from control and *Slc7a7*^*KO*^ mice. **d** Serum albumin, **e** total protein and **f** blood urea nitrogen from control and *Slc7a7*^KO^ mice. **g** Left: Representative images of α-SMA staining (brown) in kidney sections from control and *Slc7a7*^*KO*^ mice. Right: Quantification of the positive area of α-SMA staining in control and *Slc7a7*^KO^ mice. Scale bar: 250 μm. **h** Real-time RT‒PCR analysis of *Acta2* in kidney tissue. **i** High-resolution respirometry of kidney cortex tissue from control and *Slc7a7*^KO^ mice. All the data are presented as the means ± SEMs. Statistical analysis was performed via a two-tailed unpaired Student’s *t* test. Each data point represents an individual animal

Our findings indicate that a systemic factor, rather than a deficiency of *Slc7a7* in hematopoietic stem cells, underlies the erythroid differentiation defects observed in *Slc7a7* whole-body KO mice. Erythropoietin, a crucial hormone synthesized and secreted by the kidneys, is a key regulator of erythrocyte production (Goodnough et al. 2000). Intriguingly, we noted significantly decreased levels of circulating erythropoietin in *Slc7a7* whole-body knockout mice (Fig. 3a), accompanied by reduced *Epo* gene expression and protein levels in the kidney (Fig. 3b-c), which are typically associated with kidney damage and chronic kidney disease (Batchelor et al. 2020; Weiss et al. 2019). Considering the prevalence of kidney disease among LPI patients (Contreras et al. 2021), we hypothesized that *Slc7a7* deficiency might lead to kidney dysfunction, resulting in diminished erythropoietin levels and impaired RBC maturation. Confirming this hypothesis, assessments of kidney function in *Slc7a7* knockout mice revealed clear signs of early-stage chronic kidney disease, characterized by decreased serum albumin and total protein levels, elevated blood urea nitrogen levels (Fig. 3d–f) and a reduced glomerular filtration rate (Webster et al. 2017), as previously reported (Bodoy and Sotillo 2019).

We further explored potential causes of reduced erythropoietin production, such as fibrosis, by conducting histological analyses of α-SMA and Sirius Red in kidney sections. Notably, *Slc7a7* knockout mice presented increased areas of Sirius Red and α-SMA staining, the latter of which was also confirmed at the mRNA level via *Acta 2* transcript analysis (Supplementary Fig. 3a and Fig. 3h). These findings suggest that diminished kidney function is a likely mechanism for reduced erythropoietin production.

Two lines of evidence support major metabolic dysfunction in the kidneys of *Slc7a7* knockout mice. First, high-resolution respirometry revealed a substantial decrease in oxygen consumption in the renal cortex of *Slc7a7* knockout mice compared with controls (Fig. 3i), despite no changes in the mitochondrial content (Supplementary Fig. 3b). Second, there was a drastic reduction in the expression of key glucose utilization genes (*Hk2, Pgk1,* and *Ldha*) (Supplementary Fig. 3c), with an over 90% decrease in *Hk2* mRNA levels, which was mirrored at the protein level (Supplementary Fig. 3d). These results suggest significant metabolic dysfunction and functional impairments in the kidneys of *Slc7a7* knockout mice.

### Slc7a7 deficiency leads to decreased erythropoietin synthesis independent of transcriptional events

**Fig. 4:**
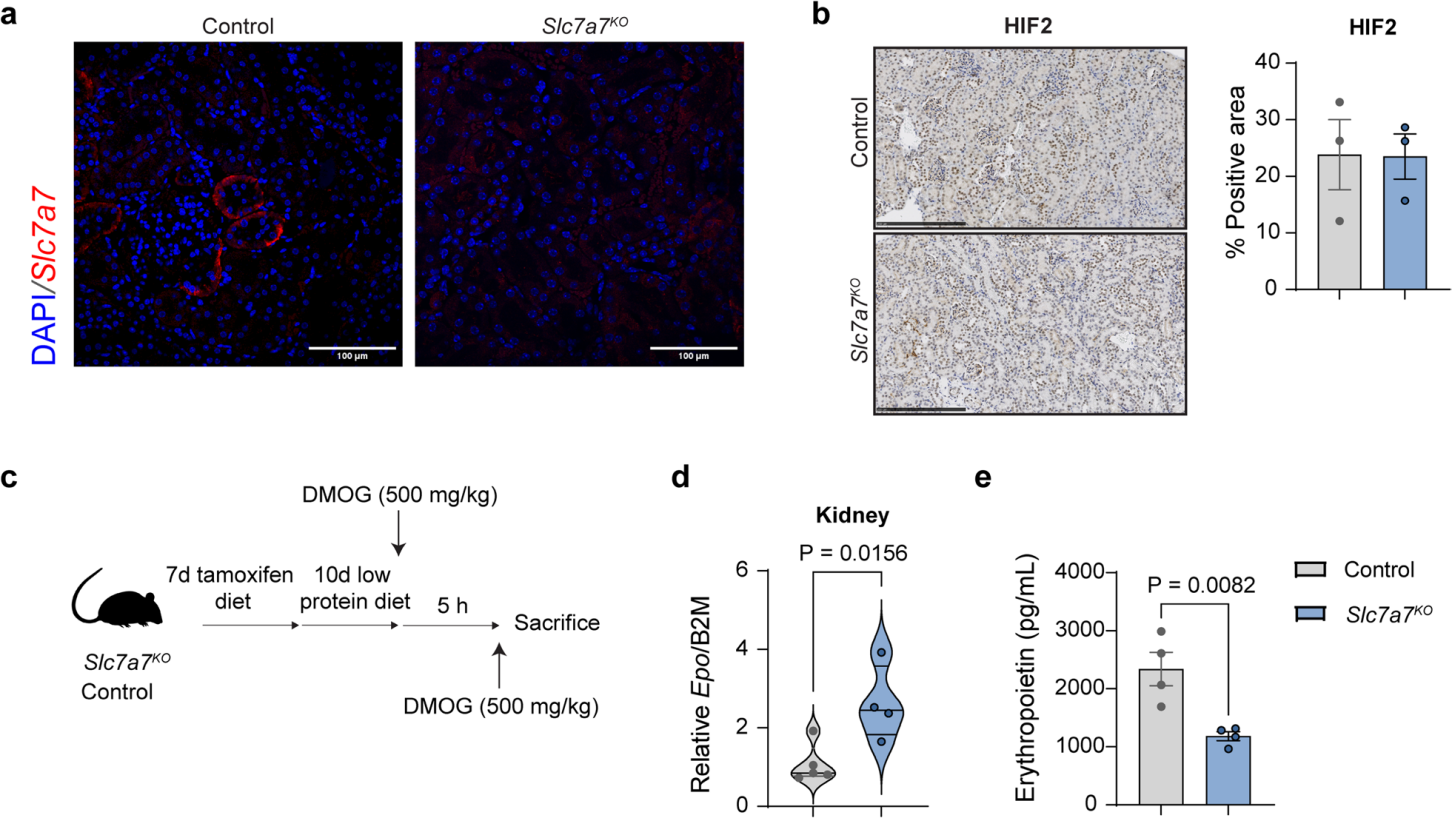
The regulation of erythropoietin production in *Slc7a7* knockout mice involves hypoxia-independent pathways. **a** Immunofluorescence staining of y^+^LAT1 protein (Red, *Slc7a7*) revealed positive localization on the basolateral side of proximal convoluted tubules in kidney sections from control and *Slc7a7*^KO^ mice. DAPI (blue) was used to stain the nuclei. Scale bar: 100 μm. Representative images from 3 independent mice per genotype are shown. **b** Left: Immunohistochemical staining of HIF-2 (brown) in kidney sections from control and *Slc7a7*^*KO*^ mice. Right: Quantification of the HIF-2-positive area revealed no significant differences between genotypes. Scale bar: 250 μm. **c** Schematic representation of the experimental protocol used to evaluate the effect of dimethyloxalylglycine (DMOG, 500 mg/kg) on erythropoietin production in control and *Slc7a7*^*KO*^ mice. The mice were treated with tamoxifen for 7 days followed by a 10-day low-protein diet, with DMOG administered 5 h prior to sacrifice. **d** Real-time RT‒PCR analysis of *Epo* gene expression in kidney tissue after DMOG administration. **e** Serum erythropoietin concentrations in control and *Slc7a7*^*KO*^ mice after DMOG treatment. All the data are presented as the means ± SEMs. Statistical analysis was performed via a two-tailed unpaired Student’s *t* test. Each data point represents a single animal

Given that *Slc7a7* is expressed in proximal tubule cells (Bauch et al. 2003) (Fig. 4a) and that renal erythropoietin-producing cells (REPCs and/or Norn cells) are closely located to tubule epithelial cells (Kobayashi et al. 2022), we next wondered whether *Slc7a7* is specifically expressed in REPCs. Using publicly available single-cell RNA sequencing datasets (Kragesteen et al. 2023), we verified that *Slc7a7* is expressed in REPC (Supplementary Fig. 4a). Next, to explore the potential molecular mechanism underlying reduced erythropoietin production, we investigated the expression of hypoxia-inducible factor-2 (HIF-2), the critical transcription factor involved in *Epo* transcriptional regulation (Kobayashi et al. 2022). Surprisingly, our analysis revealed similar levels of HIF-2 in histological preparations (Fig. 4b). Hypoxi-probe staining also revealed comparable oxygenation in both groups (Supplementary Fig. 4b), suggesting that decreased erythropoietin production is unlikely to be due to altered HIF-2 levels caused by tissue hypoxia.

Interestingly, inhibiting prolyl hydroxylases (Fig. 4c), which control HIF stability under normoxic conditions (Haase 2011), significantly increased *Epo* expression in the kidneys of knockout mice (Fig. 4d), although circulating erythropoietin levels remained notably lower (Fig. 4e). These findings indicate that potential posttranscriptional modifications affect erythropoietin stability or translation through alterations in kidney function.

### Citrulline supplementation rescues defective RBC production

**Fig. 5:**
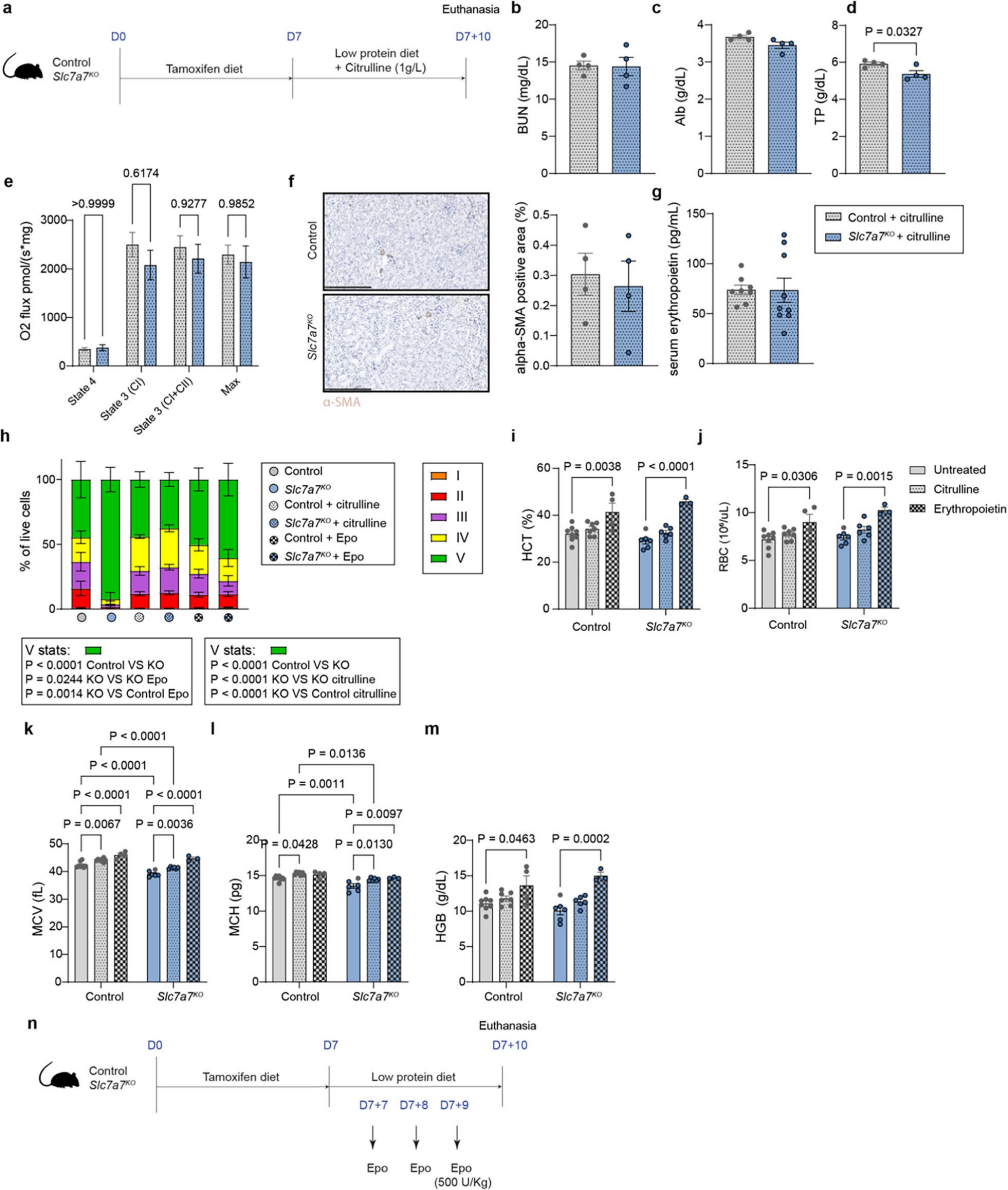
Metabolic milieu correction by citrulline improves renal function. **a** Graphical scheme of citrulline treatment in control and *Slc7a7*^KO^ mice. **b** Blood urea nitrogen, **c** albumin and **d** total protein in serum from control and *Slc7a7* knockout mice treated with citrulline. **e** Mitochondrial respiratory capacity in mitochondrial extracts from the cortex of control and *Slc7a7*^KO^ mice as previously described. **f** Left: Representative photographs of α-SMA-stained kidney sections. Right: Quantification of the positive area of α-SMA staining in control and *Slc7a7*^KO^ mice treated with citrulline. **g** ELISA of serum erythropoietin in mice treated with citrulline. **h** Percentages of erythroblast populations in control and *Slc7a7*^KO^ mice untreated, treated with citrulline or with erythropoietin. The data are presented as the means ± SEMs. Two-way ANOVA. **i** MCV, **j** HCT, **k** MCH, **l** HGB and **m** RBC counts in the whole blood of control and *Slc7a7*^KO^ untreated mice (solid bars), control and *Slc7a7*^KO^ mice treated with citrulline (polka dots), and control and *Slc7a7*^KO^ mice treated with erythropoietin (checkered pattern). **n** Graphical scheme of erythropoietin administration in control and *Slc7a7*^KO^ mice. Unless specified, all the data are presented as the means ± SEMs. Statistical analysis was performed via a two-tailed unpaired Student’s *t* test. Each data point represents a single animal

We next asked whether restoring partially the circulation levels of arginine and hyperammonemia via citrulline treatment, as previously described (Bodoy and Sotillo 2019), could ameliorate kidney dysfunction and consequently rescue the impaired RBC maturation process (Fig. 5a). Notably, normalizing the metabolic milieu improved renal function (Bodoy and Sotillo 2019), as reflected by normalized levels of blood urea nitrogen and circulating albumin, with a trend toward normalized total protein levels in the serum (Fig. 5b–d). Additionally, kidney oxygen consumption (Fig. 5e) and histological analysis via α-SMA and Sirius Red staining were comparable to those of control mice (Fig. 5f, Supplementary Fig. 5a), suggesting that metabolic correction alleviated kidney impairments.

Following citrulline treatment, we evaluated the serum erythropoietin levels and erythrocyte maturation process. The serum erythropoietin levels were similar between the genotypes (Fig. 5g), which was correlated with a similar erythrocyte maturation process (Fig. 5h, Supplementary Fig. 5b). Despite these normalized processes, the mean corpuscular volume and mean corpuscular hemoglobin content were significantly different between control and *Slc7a7* knockout mice treated with citrulline (Fig. 5i–m), suggesting that the remaining metabolic derangement (hypolysinemia, residual hyperargininemia and hyperammonemia) might compromise full RBC maturation.

Given that persistent erythropoiesis alterations are potentially linked to underlying kidney dysfunction and reduced erythropoietin levels, as citrulline eases kidney complications and ameliorates the hematological dysfunctions, we investigated whether direct erythropoietin administration could improve RBC differentiation (Fig. 5n). To test this hypothesis, we administered erythropoietin (500 U/kg) daily for three consecutive days, which effectively restored the levels of erythropoietic precursors (Fig. 5h, Supplementary Fig. 5c) and normalized whole blood counts (Fig. 5i–m). Notably, this treatment also mitigated iron overload in the spleen (Supplementary Fig. 5d), indicating effective iron utilization. These findings provide crucial insights into the hematologic disturbances observed in *Slc7a7* knockout mice, mirroring the anemia typically observed in chronic kidney disease among patients with LPI. These findings suggest that targeted treatments that restore metabolic balance and directly supplement crucial hormones such as erythropoietin may offer significant therapeutic benefits.

## Discussion

By using different mouse models, we identified erythropoietin as a critical factor in the hematological abnormalities observed in the context of Lysine protein tolerance. Our findings demonstrate that *Slc7a7* knockout mice, which mimic LPI, exhibit impaired kidney function, likely due to disrupted cationic amino acid reabsorption in kidney and intestinal epithelial cells (Bodoy and Sotillo 2019). This kidney dysfunction substantially reduces erythropoietin production, shedding light on potential mechanisms contributing to the hematological complications observed in human LPI (Mauhin et al. 2017). Given these insights, erythropoietin supplementation has emerged as a promising adjunct therapy for patients with LPI. However, the clinical use of erythropoietin in LPI has been limited to patients in advanced stages of renal failure, typically during dialysis (Tanner et al. 2007), with no discernible improvement in hematological complications. Monitoring erythropoietin levels in the early stages of LPI may provide an opportunity to tailor personalized treatment strategies and improve outcomes for patients before irreversible damage occurs.

Erythropoiesis, the process of red blood cell maturation, primarily occurs in the bone marrow and is regulated by various cytokines, including erythropoietin. This glycoprotein, which is essential for red blood cell differentiation and proliferation (Goodnough et al. 2000), is synthesized predominantly in the kidney (Lacombe et al. 1991), where its production is regulated by hypoxia-inducible factors. In conditions such as chronic kidney disease or end-stage renal disease, erythropoietin levels are often insufficient to meet erythropoietic demands, exacerbating anemia (Webster et al. 2017; Haase 2011). Our findings indicate that erythropoiesis defects in *Slc7a7* knockout mice primarily stem from extrinsic factors related to the absence of *Slc7a7* in intestinal and kidney epithelial cells, highlighting kidney damage as a potential determinant of erythropoietin deficiency. This conclusion aligns with clinical observations in Finnish LPI patients, where kidney transplantation improves symptoms, emphasizing the critical role of renal function in disease progression (IJzermans et al. 2022). Additionally, our results do not exclude the possibility that the imbalance in circulating amino acids–characterized by an increase in neutral amino acids (such as glutamine) and a decrease in cationic ones, as observed in patients with LPI and in our mouse model (Simell 2001; Bodoy and Sotillo 2019)—may contribute to the defective erythrocyte maturation process given the relevance of amino acid metabolism in this process (Lyu et al. 2024; Bouthelier et al. 2020; Shima et al. 2006; Gonzalez-Menendez et al. 2023).

In line with the extrinsic origin of the hematopoietic defect of the *Slc7a7* knockout mouse, bone marrow transplant experiments further support the role of the extrinsic environment in driving erythropoietic defects in *Slc7a7* knockout mice. Control cells transplanted into *Slc7a7* knockout mice failed to rescue erythropoiesis defects, whereas *Slc7a7* knockout cells transplanted into control mice did not reproduce these defects or presented a reduced mean corpuscular volume. Similarly, specific ablation of *Slc7a7* in the myeloid lineage (Lyz2cre) and in erythroblastic precursors (ER-Cre) resulted in a negligible phenotype of bone marrow erythropoiesis. These findings strongly suggest that *Slc7a7*-mediated cationic amino acid transport is unnecessary for red blood cell maturation, in stark contrast to other related arginine transporters, such as CAT1 (Shima et al. 2006). Collectively, these results highlight the dominance of systemic metabolic and environmental factors over intrinsic cellular mechanisms in contributing to the erythropoietic defects observed in Slc7a7 knockout mice.

As previously reported, citrulline supplementation restores partially arginine amino acid plasma levels and hyperammonaemia in *Slc7a7* knockout mice. The recovery of kidney function, circulating erythropoietin levels, and erythropoiesis following citrulline supplementation observed in this study underscore systemic metabolic derangements as the primary drivers of erythropoietic defects in LPI mice. Importantly, erythropoietin supplementation also effectively restored normal red blood cell production in both control and *Slc7a7* knockout mice despite not directly addressing the underlying metabolic imbalance. These results suggest that erythropoietin supplementation serves as a valuable therapeutic option for managing anemia associated with LPI. It is worth to mention that exogenous recombinant erythropoietin is a well-established therapeutic agent for the treatment of anemia, particularly in patients with end-stage renal disease. However, its use at high doses, in some cases, is associated with hypertension and thrombosis, as a result, erythropoietin treatment should be carefully monitored (Vaziri and Zhou 2008).

While citrulline supplementation addresses the metabolic derangements underlying erythropoietic defects, its potential adverse effects, including overactive immune responses and hyperreactivity (Ogier de Baulny et al. 2012), warrant caution. In contrast, erythropoietin supplementation bypasses these risks and directly supports erythropoiesis, offering a safer and more targeted approach for managing anemia in LPI patients. Together, these strategies highlight the importance of tailoring therapeutic interventions to the specific needs of LPI patients, particularly in balancing metabolic correction with erythropoiesis support.

## Conclusions

This study provides the first evidence that metabolic derangements resulting from the absence of *Slc7a7* expression are the primary drivers of impaired kidney function, leading to reduced erythropoietin production, defective erythropoiesis, and consequent iron overload. Our findings underscore the critical role of systemic metabolic homeostasis in maintaining erythropoietic balance and iron metabolism. Importantly, this work highlights erythropoietin supplementation as a promising therapeutic strategy to address the severe hematological complications associated with lysinuric protein intolerance. By targeting underlying metabolic and hematological defects, erythropoietin therapy could significantly improve clinical outcomes and reduce disease-associated morbidity and mortality in lysinuric protein intolerance patients. These results pave the way for further studies to optimize treatment strategies and personalize therapeutic interventions for this rare and complex disorder.

## Supplementary Information

Additional file 1

Additional file 2

## Data Availability

No datasets were generated or analysed during the current study.

## Abbreviations

BM: Bone Marrow
CAA: Cationic Amino Acids
HB: Hemoglobin
HCT: Hematocrit
HIF-2: Hypoxia-Inducible Factor-2
LPI: Lysinuric Protein Intolerance
MCH: Mean Corposcular Hemoglobin
MCV: Mean Corposcular Volume
RBC: Red Blood Cell
REPCs: Renal erythropoietin-producing cells
ROS: Reactive Oxigen Species
α-SMA: Alpha Smooth Muscle Actin
SEM: Standard Error of the Mean
PBS: Phosphate Buffered Saline
