# Supplementary material for: Defective Slc7a7 transport reduces erythropoietin compromising erythropoiesis
Source: Mol Med. 2025 Jan 29;31:29. doi: 10.1186/s10020-025-01100-0 (PMC11776305; doi:10.1186/s10020-025-01100-0)
Supplement: Supplementary file 1 — Additional file 1 [file 10020_2025_1100_MOESM1_ESM.docx]

**Supporting Information for**

**Defective *Slc7a7* transport reduces erythropoietin compromising erythropoiesis**

Judith Giroud-Gerbetant^1,2^, Fernando Sotillo^1^, Gonzalo Hernández^3^, Irene Ruano^1^, David Sebastian^4,7^, Joana Fort^1,2^, Mayka Sánchez^3^, Günter Weiss^5^, Neus Prats^1^, Antonio Zorzano^1,6,7^, Manuel Palacín^1,2*^ and Susanna Bodoy^1,2,8*^

^1^Institute for Research in Biomedicine (IRB) Barcelona, The Barcelona Institute of Science and Technology (BIST), Barcelona, Spain; ^2^Centro de Investigación Biomédica en Red Enfermedades Raras (CIBERER), Barcelona, Spain; ^3^Department of Basic Sciences, Iron metabolism: Regulation and Diseases Group. Faculty of Medicine and Health Sciences. Universitat Internacional de Catalunya (UIC), Sant Cugat, Spain; ^4^Department of Biochemistry and Physiology, School of Pharmacy and Food Science, University of Barcelona, Barcelona, Spain; ^5^Department of Internal Medicine II (Infectious Diseases, Immunology, Rheumatology and Pneumology), Medical University of Innsbruck, Innsbruck, Austria; ^6^Department of Biochemistry and Molecular Biomedicine, University of Barcelona, Barcelona, Spain; ^7^Centro de Investigación Biomédica en Red Diabetes y Enfermedades Metabólicas (CIBERDEM), Barcelona, Spain; ^8^Biosciences Department, Faculty of Sciences, Technology and Engineering, University of Vic – Central University of Catalonia (UVic-UCC), Vic, Spain.

* Susanna Bodoy, Institute for Research in Biomedicine Barcelona, Baldiri Reixac, 12, Barcelona 08028, Spain; Manuel Palacín, Institute for Research in Biomedicine Barcelona, Baldiri Reixac, 12, Barcelona 08028, Spain.

**Email:** [susanna.bodoy@irbbarcelona.org](mailto:xxxxx@xxxx.xxx), [manuel.palacin@irbbarcelona.org](mailto:manuel.palacin@irbbarcelona.org).

**This file includes:**

Supplementary Figures 1-5

Supplementary Methods

**Supplementary Figures**


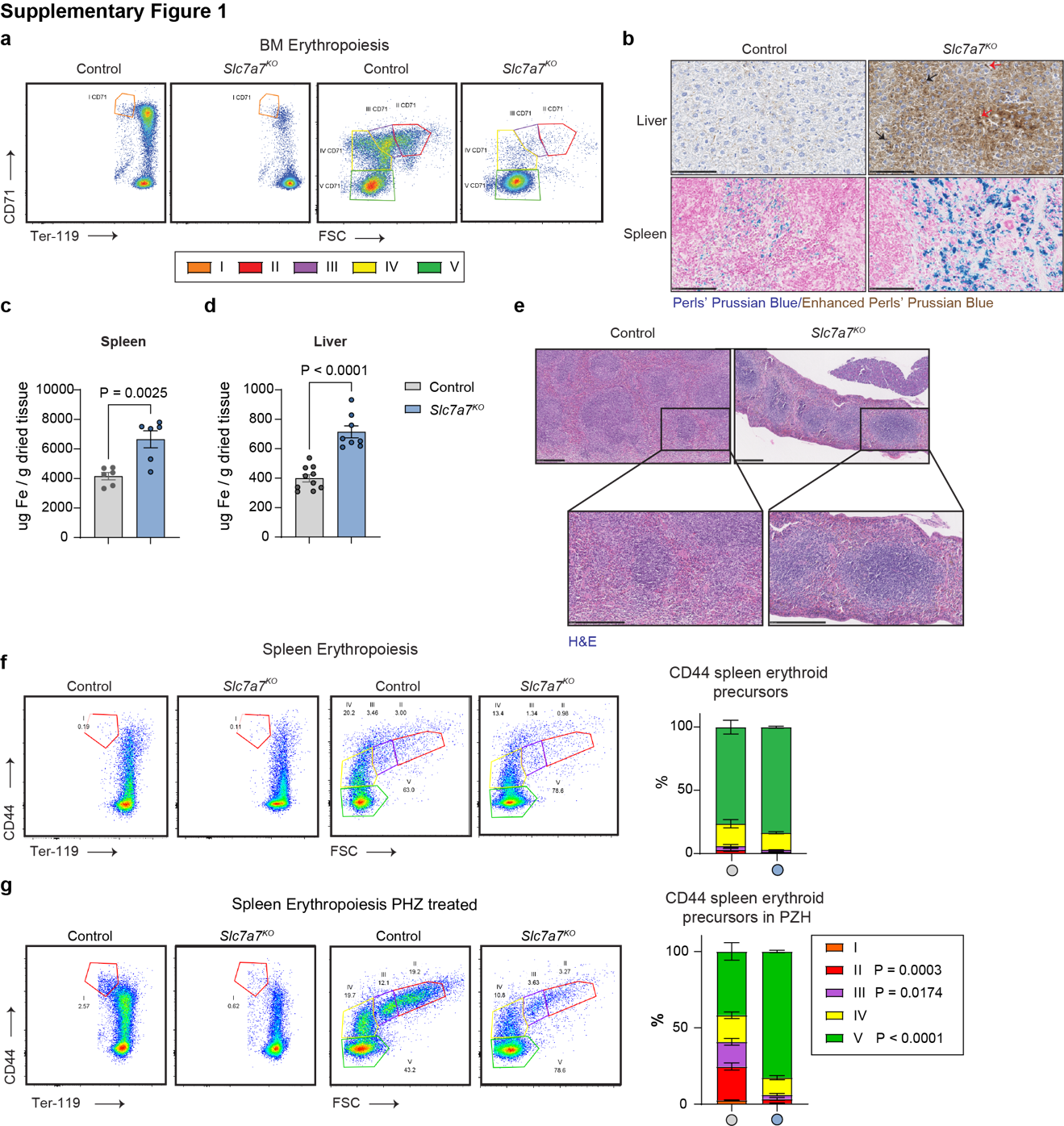


**Supplementary Figure 1. (a)** Flow cytometric analysis of bone marrow (BM) erythroid precursors from control and *Slc7a7^KO^* mice. Representative dot plots show the gating strategy for erythroid progenitors at distinct stages of maturation (I–V). Statistical significance of differences in progenitor populations between genotypes is indicated as follows: Stage V (P < 0.0001), Stage IV (P < 0.001), Stage III (P < 0.01), Stage II (P < 0.05), and Stage I (P > 0.9). The data are presented as the means ± SEMs and were analyzed via two-way ANOVA. Representative results from four independent experiments are shown. **(b)** Upper panel: Representative Enhanced Perls’ Prussian Blue staining of liver sections. Iron deposits are indicated in hepatocytes (brown staining, black arrow) and Kupffer cells (red arrow). Lower panel: Representative Perls’ Prussian Blue staining of spleen sections with hematoxylin as a background stain. Scale bars: 100 μm. **(c-d)** Iron quantification: Non-heme iron content in spleen **(c)** and liver **(d)** was quantified using the bathophenanthroline method, showing significantly elevated iron levels in *Slc7a7^KO^* mice compared to controls. Data are presented as μg Fe/g of dried tissue (mean ± SEM). **(e)** Representative hematoxylin and eosin staining of spleen sections reveals reduced cellularity and disrupted red pulp structure in *Slc7a7^KO^* mice.
**(f)** Left: Representative dot plots showing the gating strategy for splenic erythroid progenitors (Stages I–V) from control and *Slc7a7^KO^* mice. Statistical analysis indicates no significant differences in progenitor populations (Stage V: P = 0.129; Stage IV: P = 0.6368; Stage III: P = 0.9909; Stage II: P = 0.9957; Stage I: P > 0.9). Right: Bar graph showing the percentages of erythroid populations analyzed (n = 3 per group). Data are expressed as mean ± SEM, and statistical analysis was performed using a two-tailed ANOVA.
**(g)** Left: Representative dot plots show the gating strategy for erythroid progenitors in control and *Slc7a7^KO^* mice after phenylhydrazine administration. Statistical significance is indicated for each stage: Stage V (P < 0.0001), Stage IV (P = 0.5430), Stage III (P = 0.0174), Stage II (P = 0.0003), and Stage I (P = 0.9950). Right: Bar graph showing the percentages of erythroid populations analyzed (n = 5 for controls, n = 3 for *Slc7a7^KO^* mice). Data are expressed as mean ± SEM, and statistical analysis was performed using a two-tailed ANOVA.

Data information: Unless specified, all data are expressed as mean ± SEM, with statistical analysis performed using a two-tailed unpaired Student’s t-test. Each data point represents an individual animal.


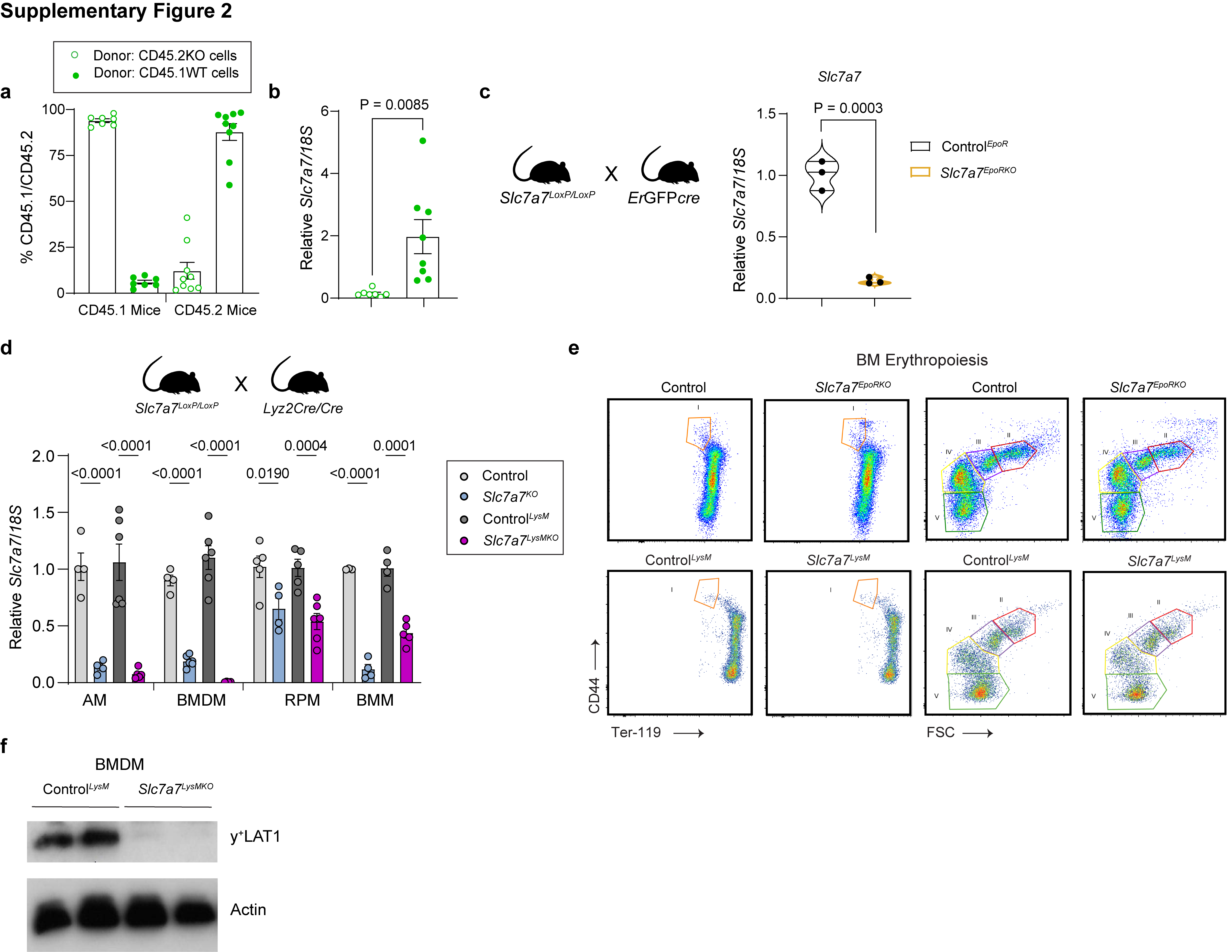


**Supplementary Figure 2. (a)** Percentage of engrafted cells in the peripheral blood of CD45.1 control mice transplanted with CD45.2 *Slc7a7^KO^* cells and CD45.2 *Slc7a7^KO^* mice transplanted with CD45.1 control cells, analyzed by flow cytometry, demonstrating efficient chimerism. **(b)** Real-time RT-PCR analysis of *Slc7a7* expression in total BM from Cd45.1 mice transplanted with Cd45.2 *Slc7a7^KO^* cells, and Cd45.2 mice transplanted with Cd45.1 control cells, confirming reduced *Slc7a7* expression in knockout-transplanted recipients. **(c)** Real-time RT-PCR analysis of *Slc7a7* expression in sorted erythroblasts from control and *Slc7a7^EpoRKO^* mice. **(d)** Real-time RT-PCR analysis of *Slc7a7* in isolated alveolar macrophages (AM), bone marrow-derived macrophages (BMDM), red pulp macrophages (RPM) and bone marrow macrophages (BMM) from *Slc7a7^KO^*, *Slc7a7^LysMKO^* mice and its respectable controls. **(e)** Flow cytometry analysis of BM erythroid precursors (Stages I–V) from *Slc7a7^LysMKO^*, *Slc7a7^EpoRKO^*, and their respective controls. Representative dot plots display the gating strategy for erythroid progenitors at various maturation stages (I–V), showing no significant differences among genotypes. **(f)** Western blot analysis of y^+^LAT1 protein levels in BMDMs from *Slc7a7^LysMKO^* and control mice. y^+^LAT1 protein is undetectable in knockout macrophages, confirming the effective ablation of *Slc7a7*. Actin was used as a loading control. Data Information: Data are presented as mean ± SEM. Statistical analysis was performed using a two-tailed unpaired Student’s t-test. Each data point represents a single animal.

**Supplementary Figure 3. (a)** Left panel: Representative images of Picrosirius Red-stained kidney sections from control and *Slc7a7^KO^* mice, highlighting increased collagen deposition in the knockout kidneys. Right panel: Quantification of the collagen-positive area indicates a significant increase in fibrosis in *Slc7a7^KO^* mice.
**(b)** Relative mitochondrial DNA content in kidney samples from control and *Slc7a7^KO^* mice, showing no significant differences between genotypes. **(c)** Real-time RT-PCR analysis of key metabolic genes in total kidney homogenates. Transcript levels of *Hk2* (hexokinase 2), *Pgk1* (phosphoglycerate kinase 1), and *Ldha* (lactate dehydrogenase A) are significantly reduced in *Slc7a7^KO^* kidneys compared to controls, suggesting impaired glucose metabolism. **(d)** Western blot analysis of HK-2 protein expression in kidney cortex samples from control and *Slc7a7^KO^* mice. A marked reduction in HK-2 protein levels is observed in *Slc7a7^KO^* kidneys, with GAPDH used as a loading control. Data Information: Data are presented as mean ± SEM. Statistical analysis was performed using a two-tailed unpaired Student’s t-test. Each data point represents an individual animal.


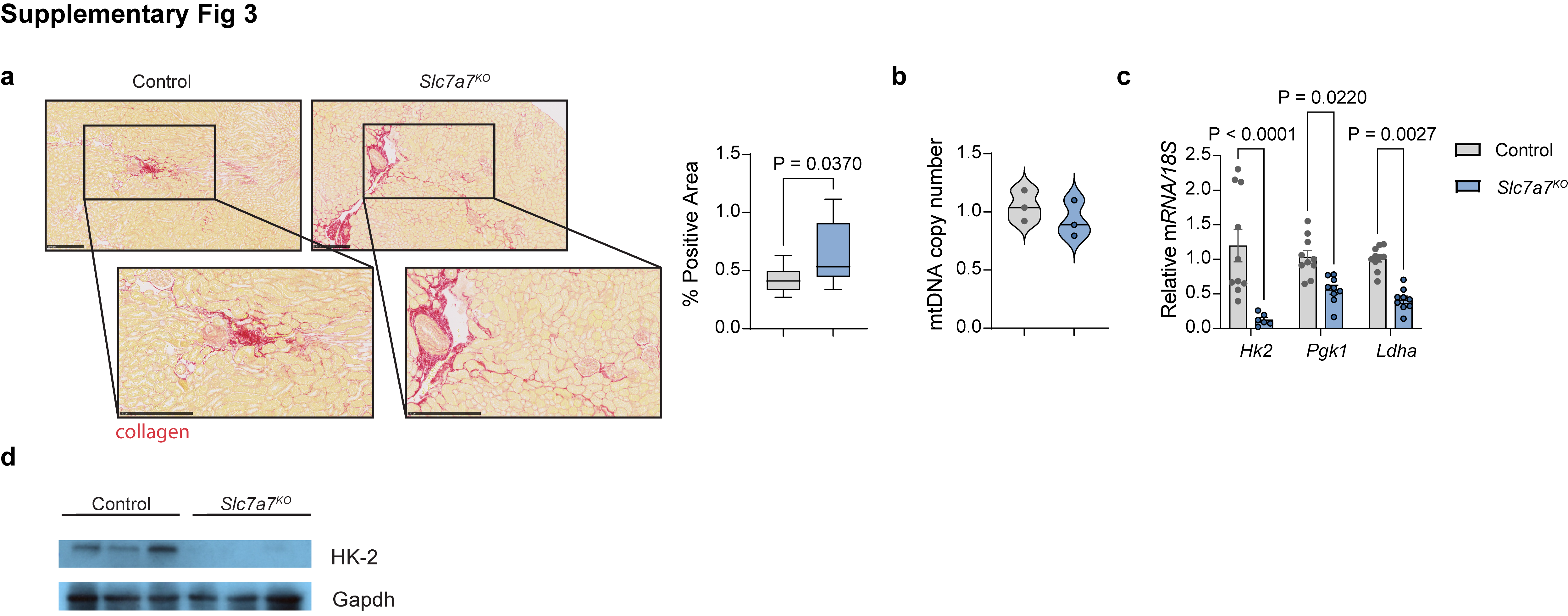


**
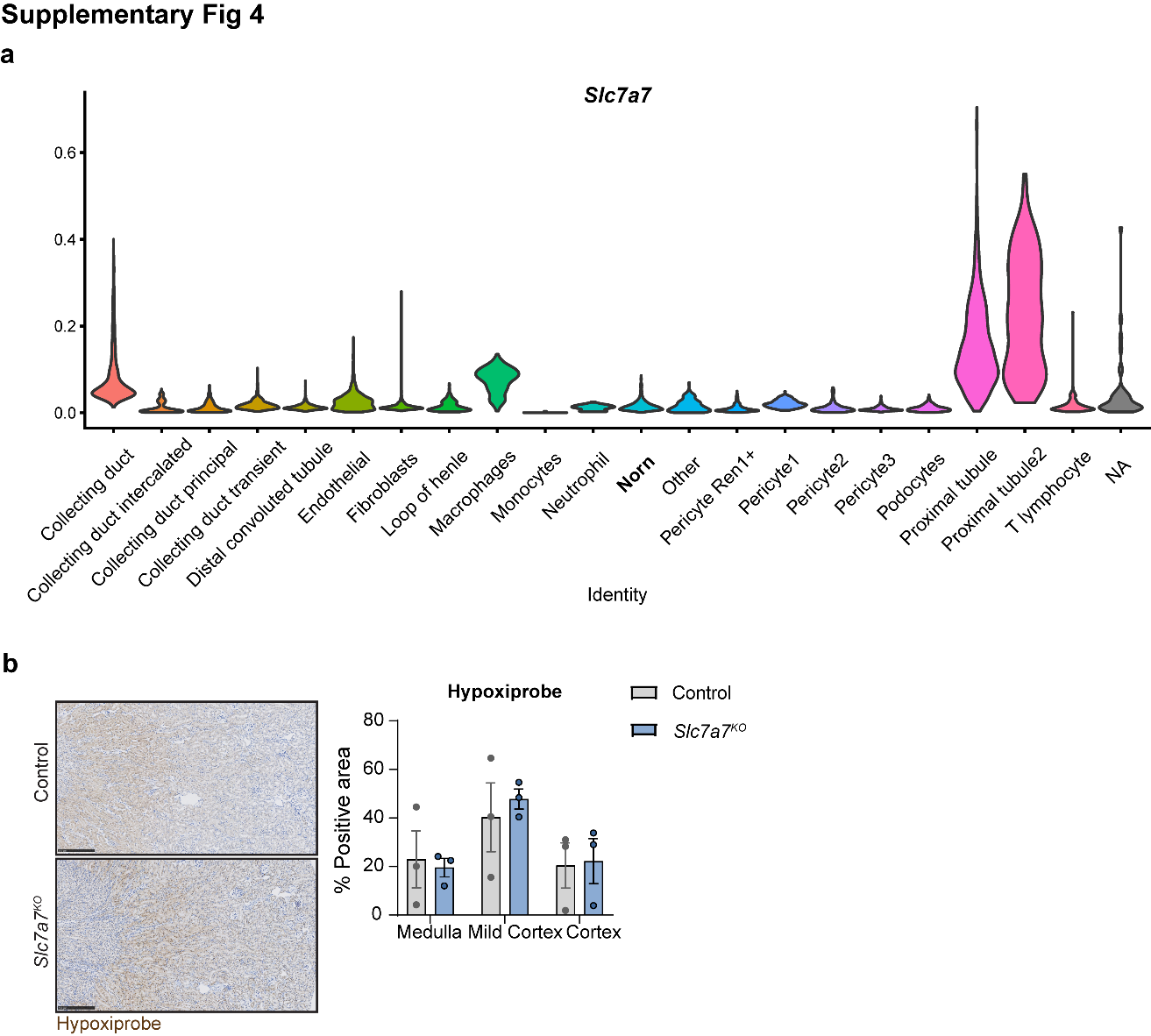
**

**Supplementary Figure 4. (a)** Violin plot showing *Slc7a7* expression across distinct kidney cell populations based on publicly available single-cell RNA sequencing data [1]. **(b)** Representative images n=6 per genotype of hypoxyprobe-stained kidney sections from control and *Slc7a7^KO^* mice. Quantification of the hypoxyprobe-positive area indicates no significant differences in tissue oxygenation levels between control and knockout mice across the medulla, mid-cortex, and cortex regions. Data are expressed as mean ± SEM, and statistical analysis was performed using a two-tailed unpaired Student’s t-test.


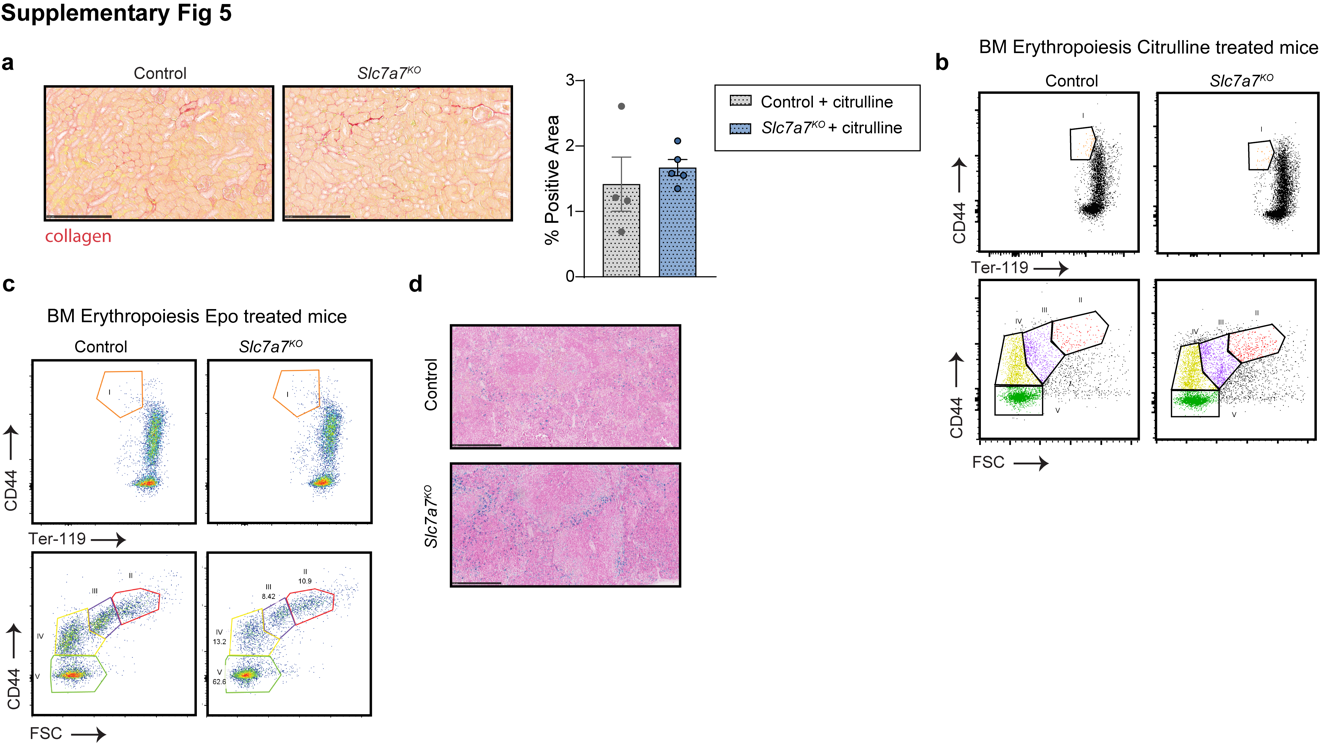


**Supplementary Figure 5. (a)** Left panel: Representative images of Picrosirius Red staining in kidney sections from control and *Slc7a7^KO^* mice supplemented with citrulline (1 g/L). Right panel: Quantification of collagen-positive areas, showing no significant differences between control and knockout mice with citrulline treatment. **(b)** Flow cytometric analysis of BM erythroid precursors (Stages I–V) in control and *Slc7a7^KO^* mice supplemented with citrulline. Representative dot plots depict the gating strategy used for erythroid progenitor identification. **(c)** Flow cytometry analysis of BM erythroid precursors in control and *Slc7a7^KO^* mice following three days of erythropoietin administration. Representative dot plots illustrate erythroid maturation stages (I–V). **(d)** Left panel: Representative images of Perls’ Prussian Blue staining in spleen sections from control and *Slc7a7^KO^* mice treated with erythropoietin (n = 7 per genotype). Images highlight reduced iron deposition in knockout mice post-treatment. Right panel: Hematoxylin and eosin staining was used as a background to aid visualization. Scale bars: 250 μm.

Data Information: Data are presented as mean ± SEM. Statistical analysis was performed using a two-tailed unpaired Student’s t-test. Each data point represents an individual animal.

**Supplementary methods**

**Animals**

*Slc7a7^loxP/loxP^* mice were generated by Eurogentec. To generate the *Slc7a7* knockout model, *Slc7a7^loxP/loxP^* animals were crossed with UBC-Cre-ERT2 mice from The Jackson Laboratory [2]. Mice were housed in groups of 2–5 per cage and were kept under a 12 h dark-light period. Food and water were supplied *ad libitum*. *Slc7a7*^loxP/loxP^ (Slc7a7LoxP/LoxP and UBC-Cre-ERT2+/-) and control (Slc7a7LoxP/LoxP not bearing UBC-Cre-ERT2 protein) mice were fed a standard diet (Teklad global 14% protein rodent maintenance diet) until tamoxifen induction, which consisted of a tamoxifen diet for one week. After the induction period, *Slc7a7* ^loxP/loxP^ and control mice were maintained on a low-protein diet for 7–10 days prior to sacrifice. Control and *Slc7a7* knockout littermates on a C57Bl6/J genetic background were sacrificed at 10–12 weeks of age by cervical dislocation. Tissues were dissected and flash-frozen in liquid nitrogen for RNA, protein, and iron quantification studies. For hematological and biochemical studies, blood was collected from a cardiac puncture in tubes containing either EDTA or heparin. The bone marrow was flushed from femur and tibia bones. For experiments, males and females were indistinguishably used.

Macrophage-specific and RBC-specific *Slc7a7* loss of function (*Slc7a7^LysMKO^*, *Slc7a7^EpoRKO^*) mice were generated by breeding mice with *Slc7a7^loxP/loxP^* mice with LysM-Cre (a gift from Dr. Nebreda’s group at IRB Barcelona, Barcelona, Spain) on a C57Bl6/J genetic background, and EpoR-cre (a gift from Dr. Klingmuller group at Max-Planck-Institute, Freiburg, Germany), respectively. Control mice were not bearing the corresponding LysM-Cre or EpoR-Cre. For experiments, males and females were indistinguishably used. *Slc7a7^EpoRKO^* mice are on a mixed background (BALB - C57Bl6/J).

Control and Slc7a7 knockout mice were intraperitoneally injected with recombinant human erythropoietin (500 U/kg/day; R&D Systems) daily for three consecutive days. Mice in the control group were injected with an equivalent amount of saline solution. For phenylhydrazine (Merck Life Science, 114715) treatment, mice were injected with 40 μg/g body weight of PHZ 48 h prior to sacrifice. For dimethyloxalyglycine (DMOG, 400091-50MG), 500 mg/kg of mice were injected 5 hours prior to sacrifice. One hour before, a second dose of 500 mg/kg per mice was injected.

**Bone Marrow Transplant experiments**

Recipient mice were lethally irradiated (9.5Gy) and transplanted with 2x10^6^ bone marrow cells by retro-orbital injection [3]. For the re-population experiments, total bone marrow cells from either *Slc7a7* knockout (CD45.2) or control (CD45.1) mice were transplanted into lethally irradiated B6 recipient mice (CD45.1 or CD45.2). As a follow-up step, five weeks after transplantation to allow whole body hematopoiesis regeneration, mice were subjected to tamoxifen diet for 7 days and then treated with a low-protein diet for 10 days prior to the sacrifice. Bone marrow reconstitution was monitored by flow cytometry. γ-Irradiation of mice was performed in a 137Cs-γ IBL 437C H irradiator (Shering CIS bio international) at 2.56Gy/min rate for the indicated dosage. The irradiated mice were inspected daily. Mice were given Baytril water containing antibiotics (Bayer, Shawnee Mission, JS) for at least 30 days to reduce the probability of infection from opportunistic pathogens

**Colony Forming Units Assay (CFUs)**

12500 freshly sorted Lin^-^Sca^-^1^+^cKit^+^ cells were isolated from femur and tibiae from control and *Slc7a7* knockout mice and cultured in MethoCult M3434 (StemCell technology). The number and morphology of the colonies was assessed with phase contrast microscope after 8 days of culture.

**Mitochondrial DNA content**

Genomic DNA was extracted from kidney tissue using DNeasy Blood and Tissue kit (Qiagen, 69504) following the manufacturer’s instructions. Mitochondrial and nuclear DNA content were assessed by measuring 16s, COX2 and HK2, UCP2 respectively by real-time PCR platform (Applied Biosystems) and the SYBRc® Green PCR Master Mix. The following primers were used: mouse 16s forward 5’- CCGCAAGGGAAAGATGAAAGAC-3’ and reverse 5’- TCGTTTGGTTTCGGGGTTTC-3’, mouse cox2 forward 5’- GTTGATAACCGAGTCGTTCTGC-3’ and reverse 5’-CCTGGGATGGCATCAGTTTT-3’, mouse Hk2 forward 5’-TCTGGCTCTGAGATCCATCTTCA-3’ and reverse 5’-CCGGCCTCTTAACCACATTCC-3’, mouse Ucp2 forward 5’-CTACAGATGTGGTAAAGGTCCGC-3’ and reverse 5’-GCAATGGTCTTGTAGGCTTCG-3’.

**Protein analysis**

Protein extraction and quantification, and western blotting procedures were performed as described previously [4]. Hexokinase 2 antibody was purchased form Cell Signaling Technology (Ref: 2106S) and used at a 1:1000 dilution. Gapdh antibody was also obtained from Cell Signaling Technology (Ref: 2118L). Western blotting bands were quantified using ImageJ software.

**Serum measurements**

Commercial enzyme-linked immunosorbent assay kits were used to determine erythropoietin (R&D Systems, MEP00B) proteins in fresh serum.

**RNA extraction and quantitative real-time PCR**

Mice were killed by cervical dislocation, and tissues were immediately frozen for RNA isolation. Total RNA was isolated from purified cells using magnetic beads and the Agencourt RNA Clean XP Kit (Beckman Coulter). Quality and quantity were assessed using a Bioanalyzer 2100. RNA was amplified for 22 cycles and purified at the IRB Functional Genomic Facility using the PureLink Quick PCR Purification kit (Invitrogen). Amplification was performed using the ABI Prism 7900 HT real-time PCR platform (Applied Biosystems) and the SYBRc® Green PCR Master Mix. Gene expression levels were normalized with *18S* as housekeeping gene. The primer sequences are as follows: mouse *Slc7a7* forward 5’-TCAACAGCACCAAGTATGAAGTG-3’ and reverse 5’- AGCCCAGATGACCAGTGAGA-3’ mouse *18S* forward 5’-GTAACCCGTTGAACCCCATT-3’ and reverse 5’-CCATCCAATCGGTAGTAGCG-3’, mouse *Epo* forward 5’-TGGTCTACGTAGCCTCACTTCACT-3’ and reverse 5’-TGGAGGCGACATCAATTCCT-3’, *Acta-2* forward 5’- GTCCCAGACATCAGGGAGTAA-3’ and reverse 5’- TCGGATACTTCAGCGTCAGGA-3’, and mouse *Ldha* forward 5’- TGTCTCCAGCAAAGACTACTGT-3’ and reverse 5’- GACTGTACTTGACAATGTTGGGA-3’ and mouse *Pgk1* forward 5’- ATGTCGCTTTCCAACAAGCTG-3’and reverse 5’- GCTCCATTGTCCAAGCAGAAT-3’.

For visualizing *Slc7a7* expression in Norn Cells, we used the count matrix and cell annotation from previously published data[5] was downloaded from GEO, accession number GSE193321. Data normalization and variance stabilization was performed using the SCTtransform function from the R Seurat package (v.4.3.0)[6] after removing ribosomal genes. Expression was imputed and denoised using the MAGIC algorithm as implemented in the Rmagic package [7].

**Histological sample preparation and analysis**

Liver, kidney, and spleen samples were fixed with neutral buffered formalin overnight at 4ºC. All samples were embedded in paraffin. Paraffin-embedded tissue sections (2–3-μm thickness) were air-dried and further dried at 60ºC overnight.

For iron staining, paraffin-embedded tissue sections were dewaxed and stained with Iron Stain Kit to identify iron pigment using the Dako Autostainer Plus.

For the Picrosirius Red staining, tissue samples were dewax and incubated with the mordant Thiosemicarbazide 99% (Sigma, T33405) for 10 min, washed in distilled water and therefore incubated with 0.5% direct Red 80 (365548, Sigma) solution in Picric Acid Solution 1.3% (P6744-1GA, Sigma) for 90 min and finally rinsed with 1% acetic acid (Sigma, 320099) for 1 min. In all cases samples were dehydrated and mounted with Mounting Medium, Toluene-Free (CS705, Dako, Agilent) using a Dako CoverStainer.

Immunofluorescence staining of y+LAT1 in kidney sections was performed as described previously [8].

For hypoxyprobe staining, sections were dewaxed and an antigen retrieval process was done using Trisodium citrate 2-hydrate (Sigma 131655.1210) pH 6 in autoclave at 121º 20 min. Quenching of endogenous peroxidase was performed by 10 min of incubation with Peroxidase-Blocking Solution (Dako REAL S2023). Primary antibody Hydroxyprobe (Mab1) (Hypoxyprobe, Inc) was diluted 1:100 with DAKO antibody diluent (Dako – Agilent, S0809) for 60 min at room temperature. The secondary antibody used was a Polyclonal goat anti-mouse HRP (Dako, P0447) incubated 30 min. Antigen–antibody complexes were revealed with 3-3′-diaminobenzidine (K3468, Dako) with a time exposure of 7 min. Sections were counterstained with hematoxylin (Dako, S202084) and mounted with Mounting Medium, Toluene-Free (CS705, Dako) using a Dako CoverStainer.

For Hif2 staining, sections were dewaxed and the antigen retrieval process was done using Trisodium citrate 2-hydrate (Sigma 131655.1210) pH 9 using a PT Link (Agilent Technologies) at 97º 20 min. Quenching of endogenous peroxidase was performed by 10 min of incubation with Peroxidase-Blocking Solution (Dako REAL S2023). Unspecific unions were blocked using 10% of goat normal serum (Life technology 16210064) and 2.5% BSA for 45 min. Primary antibody Rabbit Polyclonal HIF-2 alpha/EPAS1 (NB100-122, Novusbio) was diluted 1:500 with EnVision FLEX Antibody diluent (k800621, DAKO, Agilent) antibody diluent (Dako – Agilent, S0809) for 120 min at room temperature.  The secondary antibody used was a BrightVision Poly-HRP-Anti Rabbit IgG Biotin-free, ready to use (catalog no. DPVR-110HRP, Immunologic) for 45 min at room temperature. Antigen–antibody complexes were revealed with 3-3′-diaminobenzidine (K3468, Dako) a time exposure of 10 min. Sections were counterstained with hematoxylin (Dako, S202084) and mounted with Mounting Medium, Toluene-Free (CS705, Dako) using a Dako CoverStainer.

Brightfield images were acquired with a NanoZoomer-2.0 HT C9600 digital scanner (Hamamatsu) equipped with a 20× objective. All images were visualized with NDP.view 2 U123888-01 software using a gamma correction set at 1.8 in the image control panel of the software.

[1] B. K. Kragesteen *et al.*, “The transcriptional and regulatory identity of erythropoietin producing cells,” *Nat Med*, Apr. 2023, doi: 10.1038/s41591-023-02314-7.

[2] S. Bodoy *et al.*, “Inducible Slc7a7 Knockout Mouse Model Recapitulates Lysinuric Protein Intolerance Disease,” *Int J Mol Sci*, vol. 20, no. 21, p. 5294, Oct. 2019, doi: 10.3390/ijms20215294.

[3] J. Farrés *et al.*, “Parp-2 is required to maintain hematopoiesis following sublethal γ-irradiation in mice,” *Blood*, vol. 122, no. 1, pp. 44–54, Jul. 2013, doi: 10.1182/blood-2012-12-472845.

[4] J. Giroud-Gerbetant *et al.*, “A reduced form of nicotinamide riboside defines a new path for NAD+ biosynthesis and acts as an orally bioavailable NAD+ precursor,” *Mol Metab*, vol. 30, pp. 192–202, Dec. 2019, doi: 10.1016/J.MOLMET.2019.09.013.

[5] B. K. Kragesteen *et al.*, “The transcriptional and regulatory identity of erythropoietin producing cells,” *Nature Medicine 2023 29:5*, vol. 29, no. 5, pp. 1191–1200, Apr. 2023, doi: 10.1038/s41591-023-02314-7.

[6] C. Hafemeister and R. Satija, “Normalization and variance stabilization of single-cell RNA-seq data using regularized negative binomial regression,” *Genome Biol*, vol. 20, no. 1, pp. 1–15, Dec. 2019, doi: 10.1186/S13059-019-1874-1/FIGURES/6.

[7] D. van Dijk *et al.*, “Recovering Gene Interactions from Single-Cell Data Using Data Diffusion,” *Cell*, vol. 174, no. 3, pp. 716-729.e27, Jul. 2018, doi: 10.1016/J.CELL.2018.05.061/ATTACHMENT/7AC4A2F9-F866-4375-960C-C4CEAAC61F07/MMC3.XLSX.

[8] M. Espino Guarch *et al.*, “Mutations in L-type amino acid transporter-2 support SLC7A8 as a novel gene involved in age-related hearing loss,” *Elife*, vol. 7, p. e31511, Jan. 2018, doi: 10.7554/eLife.31511.
