# Supplementary material for: Defective Slc7a7 transport reduces erythropoietin compromising erythropoiesis
Source: Mol Med. 2025 Jan 29;31:29. doi: 10.1186/s10020-025-01100-0 (PMC11776305; doi:10.1186/s10020-025-01100-0)
Supplement: Supplementary file 2 — Additional file 2 [file 10020_2025_1100_MOESM2_ESM.docx]

**TABLES**

| **20 days Low Protein Diet** | **Genotype** | **Hb, g/dL** | **Hct, %** | **MCH, pg** | **MCV, fL** | **RBC, 10^6^/μL** |
| --- | --- | --- | --- | --- | --- | --- |
|  | **Control** | 11.6 ± 0.17 | 34.85 ± 0.6 | 15.08 ± 0.38 | 45.33 ± 0.79 | 7.69 ± 0.17 |
|  | ***Slc7a7* knockout** | 10.53 ± 0.52 P = 0.0786 | 30.9 ± 1.15 P = 0.0218 | 13.97 ± 0.03 P = 0.0589 | 41.07 ± 0.67 P = 0.0115 | 7.6 ± 0.2 |

**Supplementary Table 1**. Whole-blood count analysis of Slc7a7 knockout mice after tamoxifen induction and 20 days of low protein diet. Hemoglobin (Hb), hematocrit (Hct), mean corpuscular hemoglobin (MCH), mean corpuscular volume (MCV), red blood cell count (RBC) and reticulocyte count in the control and Slc7a7 knockout. Data information: Data are presented as the means ± SEMs. N=8. Statistical analysis was performed via a two-tailed unpaired Student’s t test and P value are shown.
